# Navigating emotional resonance and language identity through World Englishes education: an experimental intervention with EFL learners in China

**DOI:** 10.3389/fpsyg.2026.1793431

**Published:** 2026-02-24

**Authors:** Yaoyao Zhu, Yan Zhang, Chunlin Yao

**Affiliations:** 1 School of Foreign Languages, Xinxiang University, Xinxiang, China; 2 School of Foreign Languages, Tangshan University, Tangshan, China; 3 School of Foreign Languages, Tianjin Chengjian University, Tianjin, China

**Keywords:** China, EFL learner, emotional resonance, language identity, World Englishes

## Abstract

This quasi-experimental study investigated the impact of World Englishes education on enhancing EFL learners' English language identity and their English emotional resonance in China. Data were collected via a survey comprising two questionnaires. Two teaching classes, which initially exhibited no significant differences in English language identity and emotional resonance with English, were randomly assigned as the experimental and control classes. Both classes received identical instructional content, except that the experimental class participated in 16 academic hours focused on World Englishes, whereas the control class received 16 academic hours on the history of the English language. After a period of 10 weeks, the research team conducted a follow-up survey assessing students' English language identity and emotional resonance with English. The findings indicated a greater improvement in these measures within the experimental class compared to the control class. This study thus confirms that education cantered on World Englishes significantly enhances EFL learners' English language identity and their emotional resonance to the language.

## 1 Introduction

Language functions as a conduit for conveying both semantic meaning and emotional significance (Weimer et al., 2022), exhibiting a profound relationship with empathy. When an emotional stimulus, such as a narrative, visual image, or interpersonal interaction, elicits a strong and relatable response, individuals may experience a deep, often subconscious, connection with their own emotions or values. This phenomenon is termed emotional resonance (Rühlemann, 2022). Emotional resonance is intricately linked to both language acquisition and language use. The process of acquiring a language transcends the mere mastery of pronunciation, vocabulary, and grammatical rules; it also involves understanding the emotional nuances and cultural richness embedded within the language.

Successful foreign language learning requires that learners achieve not only competence in the phonological, lexical, and syntactic dimensions of the target language but also cultivate an emotional resonance akin to that of native speakers (Yu, 2021). Nonetheless, prior research has indicated that individuals' emotional resonance with foreign or second languages differs from that experienced with their first language (Bermúdez-Margaretto et al., 2025; Dekeyser et al., 2025; Liu et al., 2022; Toivo et al., 2024; Weimer et al., 2022), attributable to a variety of factors. Caldwell-Harris (2014) and Dewaele (2004) posit that the age at which language learning commences serves as a predictor of speakers' perceived emotional intensity, as this age influences both language proficiency and frequency of use (Caldwell-Harris et al., 2012). Other scholars (Dewaele et al., 2023; Harris et al., 2006; Pavlenko, 2012) attribute differences in emotional resonance to the contexts in which languages are learned. They argue that first languages are typically acquired within familial settings, where parents play a pivotal role in children's emotional socialization, encompassing emotional reactivity, behavioral and physiological emotion regulation, emotional knowledge, and social competence (Leerkes and Bailes, 2019). In contrast, foreign or second languages are often learned in more constrained environments, such as formal classrooms or workplaces, where learners have limited opportunities to engage with the affective connotations of the target language in authentic social contexts (Pavlenko, 2012). This is particularly salient in the case of English language learning in China, where English functions as a foreign language and learners typically have scarce opportunities to use it as a medium of daily communication (Yao, 2022). Drawing on these observations, we propose the following hypothesis: Learners' emotional resonance with the English language is at a comparatively low to medium level.

The construction of English language identity among EFL learners constitutes a significant and widely examined issue within the fields of foreign language education and language psychology. In numerous EFL contexts, learners' English language identity is frequently observed to be underdeveloped or exist at a low level. This condition is shaped by a confluence of factors: restricted opportunities for authentic language use, a predominant focus on instrumental learning objectives, and internal conflicts between the target language and one's primary linguistic and cultural identity. Empirical evidence underscores this phenomenon. For instance, research with Saudi EFL learners indicates that while English is highly valued for instrumental purposes like career advancement, this orientation often does not translate into the development of a new, integrated foreign-language identity (Alotaibi and Abahussain, 2024). This widespread instrumental view is recognized as a broader challenge in EFL pedagogy (Ngo, 2025). Furthermore, identity development is complicated by the complex negotiation between linguistic systems. Studies involving Iranian EFL learners reveal a tendency to compartmentalize language use, associating different languages with distinct emotional and functional domains, thereby impeding the formation of a unified linguistic self-concept (Abedini, 2025). In settings such as China, the scarcity of daily opportunities to employ English in meaningful social interactions hinders the affective engagement crucial for robust identity formation (Yao, 2022). Synthesizing these observations, the following hypothesis is advanced: EFL learners' language identity in relation to English in China is at a comparatively low to medium level.

Several studies (Dekeyser et al., 2025; Grosjean, 2024) have investigated the emotional resonance experienced by fluent bilingual individuals. These studies reveal that bilinguals seldom exhibit equivalent emotional resonance and proficiency across their languages, as their language use varies depending on contextual factors, interlocutors, and other considerations. Sociolinguistic research (Atkinson et al., 2025; Eastman, 1985; Yao and Zuckermann, 2016) has demonstrated a reciprocal influence between language identity and language use. Compared to other determinants affecting learners' emotional resonance with a foreign language, such as the age of language acquisition and learning environments, language identity appears to be more amenable to change. Consequently, enhancing the English language identity of learners acquiring English as a foreign language represents a feasible and promising strategy to foster their emotional resonance with the language. Given that cultivating emotional resonance with the target language is a critical objective in foreign language education, strengthening English language identity may also contribute to improving the overall effectiveness of foreign language instruction.

English functions as a global lingua franca across academia, education, economics, and international communication, and it is studied extensively worldwide. China, in particular, hosts a substantial population of English learners. Nearly all students in China, from as early as grade three in primary school (and even grade one in metropolitan areas) through to postgraduate studies, are required to learn English as a foreign language (Yao, 2022). According to the *Language Situation in China: Annual Report (2022)*, by the end of 2021, the number of English learners in China exceeded 400 million (Guo, 2022). Despite this widespread engagement, the quality of English education in China falls short of official standards. Reports indicate that fewer than 30% of English learners successfully pass the national English proficiency examination (Yao, 2022). The factors contributing to this suboptimal educational quality are multifaceted. However, a primary issue is the perception among both teachers and learners that English is a foreign language belonging to others (Zhang and Yao, 2025). This perception undermines learners' confidence and weakens their linguistic identity with respect to English (Cao et al., 2024).

In recent years, the paradigm of World Englishes (often synonymous with Global Englishes) has gained significant scholarly traction. This paradigm fundamentally acknowledges the linguistic diversity and legitimacy of English varieties that emerge from its global use across distinct sociocultural contexts (Rose and McKinley, 2025). It posits that established varieties such as American or British English, alongside emerging ones like Chinese English, are equally valid. Consequently, English is no longer conceptualized as the exclusive property of its traditional native speakers; instead, all users, regardless of geographical or cultural origin, are recognized as legitimate owners of the language. Empirically, interventions incorporating World Englishes into curricula have shown positive outcomes. For instance, research in the Chinese higher education context indicates that such pedagogical approaches can effectively bolster students' confidence in their spoken English (Cao et al., 2024). Theoretically, exposure to this pluralistic view of English is argued to strengthen learners' identification with the language and enhance their affective engagement. Synthesizing this empirical and theoretical foundation, we advance the following hypotheses: (1) World Englishes education promotes Chinese EFL learners' English language identity. (2) World Englishes education promotes Chinese EFL learners' emotional resonance with English.

Based on the extensive literature reviewed, the present study seeks to empirically investigate the impact of pedagogical interventions grounded in the World Englishes paradigm on Chinese EFL learners' English language identity and their emotional resonance with the English language. Specifically, this research aims to verify the following three hypotheses.

Hypothesis 1: Chinese EFL learners' emotional resonance and language identity in relation to English are at a comparatively low to medium level.

Hypothesis 2: World Englishes education promotes Chinese EFL learners' English language identity.

Hypothesis 3: World Englishes education promotes Chinese EFL learners' emotional resonance with English.

## 2 Methodology

This quasi-experimental study received approval from the Committee of Ethics and Integrity in Research with Humans at the S Institute (a pseudonym employed here to address ethical considerations). It employs a pre-test/post-test control group approach to examine the effects of World Englishes instruction on learners' English language identity and emotional resonance. The choice of this design is guided by the research objectives, which focus on assessing the extent and nature of the intervention's impact. Given the interest in evaluating causal relationships, an experimental framework allows for the systematic manipulation of the independent variable (the World Englishes curriculum) while controlling for external influences through the use of a comparison group. The design further supports quantitative measurement at multiple time points, enabling statistical analysis of change over time. This approach balances methodological rigor with practical feasibility in intact classroom settings, thereby strengthening the validity of claims regarding the influence of the pedagogical intervention while maintaining relevance to authentic educational contexts.

### 2.1 Participants

English language instruction in China is delivered not only within formal educational settings but also through private training institutions. This study was conducted at the S Institute, a corporate entity specializing in English language education, which enrolls over 1,000 learners annually in its training programs. Participants were recruited from this institute. A simple random sampling procedure was employed for participant selection. First, four out of eight existing instructional classes were randomly chosen to assess students' English language identity and emotional resonance. Following comparative analysis, two classes demonstrating no significant differences on these baseline measures were randomly designated as the experimental class (EC) and the control class (CC). All participants were native Chinese speakers. The EC comprised 38 students (24 females, 14 males), aged 18–20 years, with a mean age of 18.6. The CC included 39 students (22 females, 17 males), aged 18–21 years, with a mean age of 19.2.

### 2.2 Instruments

Data were collected using two questionnaires. The first instrument, the *English Language Identity Questionnaire*, was adapted from Khatib and Rezaei (2013). Beyond demographic items, it contained 20 items assessing English language identity across six domains: attachment to the Chinese language (3 items), pronunciation attitudes (3 items), perceptions of language and social status (3 items), language use and exposure in society (5 items), spoken language knowledge (3 items), and knowledge and attitudes regarding written language (3 items). The questionnaire demonstrated high reliability, with a test–retest correlation coefficient (*r*) of 0.835. Its validity was confirmed by three experts, who endorsed its suitability for evaluating Chinese university students' English language identity.

The second instrument, the *Questionnaire of Learners' Emotional Resonance of English*, was adapted from a prior questionnaire (Toivo et al., 2024) by substituting references to “my L1” with “Chinese” and “my LX” with “English.” This questionnaire comprised 15 items, excluding demographic questions. Previous research (Toivo et al., 2024) reported a Cronbach's alpha of 0.867 for this instrument. In the present study, the test–retest reliability was 0.853. Three experts reviewed the questionnaire and affirmed its validity for assessing learners' emotional resonance with English.

All items in both questionnaires employed a five-point Likert scale with the following response options: “A. Completely describes me”, “B. Mostly describes me,” “C. Somewhat describes me,” “D. Slightly describes me,” and “E. Does not describe me at all.”

### 2.3 Teaching process

The instructional intervention consisted of 8-week courses delivered by a single English instructor to both the EC and the CC. Participants did not receive monetary compensation or gifts for their involvement; however, they were granted complimentary access to the courses. Prior to the intervention, all participants indicated a willingness to acquire knowledge regarding the history of the English language and World Englishes. The second and third authors provided detailed explanations of the course content to participants in both the EC and CC. Written informed consent for the publication of this report, including all associated data and images, was obtained from all participants before their enrolment.

Subsequently, the EC engaged in a course focused on World Englishes, attending eight lectures totaling approximately 16 academic hours. This course was based on the textbook *World Englishes (3rd Edition)* by Melchers and Shaw (2019) and encompassed topics such as the origins of English, its global dissemination, linguistic variation, English within the inner, outer, and expanding circles, English beyond these circles, and prospective developments of the language. Conversely, the CC participated in a course on the history of the English language, guided by the textbook *A History of English: An Introduction (2nd Edition)* by Gramley (2018). This curriculum traced the evolution of English from Old English through Middle English to Modern English. Participants in both teaching classes were assured the opportunity to attend the alternate course (World Englishes for the CC and English language history for the EC) upon completion, should they desire.

### 2.4 Data collection

Data collection commenced with the administration of questionnaires by the second and third authors to assess participants' English language identity and their emotional resonance about English. Concurrently, the English instructor employed a consistent pedagogical approach to teach English language skills, delivering the World Englishes course to the EC and the History of English course to the CC over the 8-week period. In the tenth week, the second and third authors conducted a follow-up assessment using the same questionnaires to evaluate changes in participants' English language identity and emotional resonance. Following data collection, the second and third authors performed a comprehensive analysis of the gathered data.

### 2.5 Data analysis

The second and third authors conducted an evaluation of participants' responses in the administered questionnaires. For the *English Language Identity Questionnaire*, response options A through E for items 1, 4, 5, 7, 8, 9, 10, 11, 13, 14, 18, and 20 were scored on a scale from 5 to 1, whereas for the remaining items, options A through E were scored from 1 to 5. Higher total scores corresponded to a stronger English language identity. Conversely, in the *Questionnaire of Learners' Emotional Resonance with English*, options A through E were uniformly scored from 1 to 5, with higher scores indicating greater emotional resonance with the English language. Following the scoring procedure, the second and third authors performed data analysis utilizing SPSS version 27.0. Descriptive statistics for measurement data were reported as means and standard deviations. Effect sizes were calculated using Cohen's *d*, as recommended by Ferguson (2009). Additionally, independent samples *t*-tests were employed to conduct further inferential analyses.

## 3 Results

The data collected prior to and following the intervention provide insights into the impact of World Englishes education on participants' English language identity and their emotional resonance to the English language. The subsequent data present these effects in detail.

### 3.1 Participants' English language identity and emotional resonance before intervention

Prior to the implementation of the World Englishes curriculum, baseline measurements of participants' English language identity and emotional resonance were obtained. The results for English language identity revealed closely aligned mean scores between the EC (*M* = 50.89) and the CC (*M* = 50.87) on a 100-point scale. An independent samples *t*-test confirmed no statistically significant pre-existing difference between the two groups (*p* = 0.962 > 0.05). Similarly, for emotional resonance (measured on a 75-point scale), the average scores for the EC (*M* = 39.95) and the CC (*M* = 40.18) were comparable, with the *t*-test again indicating no significant difference (*p* = 0.638 > 0.05).

These baseline scores, which approximate the midpoint of their respective scales, indicate that participants in both classes commenced the study with a moderate level of English language identity and emotional resonance. This initial finding provides empirical support for the study's first hypothesis, which posited that Chinese EFL learners' identification with and emotional connection to English would be at a comparatively low to medium level. A detailed summary of these pre-intervention measures is presented in Table 1.

**Table 1:** Emotional resonance with English and English identity before World Englishes education.

**Items**	**Class**	***M***	**S.D**.	***T***	***df***	**Sig (2-tailed)**	**Cohen's *d***	**95% Confidence interval**	
								**Lower bound**	**Upper bound**
Emotional resonance	EC	39.95	2.265	−0.473	75	0.638	−0.108	−1.210	0.745
	CC	40.18	2.037						
English identity	EC	50.89	2.064	0.048	75	0.962	0.011	−0.921	0.967
	CC	50.87	2.092						

### 3.2 Participants' English language identity after intervention

Following an 8-week instructional period, wherein the EC received a curriculum focused on World Englishes and the CC studied English history, participants' English language identity was reassessed. The results indicated a positive shift in the EC. The mean English language identity score increased from 50.89 (pre-intervention) to 52.05 (post-intervention). In contrast, scores in the CC remained relatively stable (pre-intervention: *M* = 50.87; post-intervention: *M* = 51.15).

Statistical analysis confirmed the significant impact of the World Englishes intervention. An independent samples *t*-test conducted on the post-intervention scores revealed that the EC scored significantly higher than the CC (52.05 vs. 51.15; *p* = 0.004 < 0.01; Cohen's *d* = 0.670 > 0.5, indicating a medium effect size). The analysis results supported the second hypothesis that World Englishes education promotes Chinese EFL learners' English language identity.

A detailed comparison of the post-intervention differences in English language identity between the EC and CC is presented in Table 2.

**Table 2:** Emotional resonance with English and English identity after World Englishes education.

**Items**	**Class**	***M***	**S.D**.	***T***	***df***	**Sig (2-tailed)**	**Cohen's *d***	**95% Confidence interval**	
								**Lower bound**	**Upper bound**
Emotional resonance	EC	41.13	0.963	2.429	56.992	0.018^*^	0.549	0.145	1.503
	CC	40.31	1.880						
English identity	EC	52.05	0.985	2.957	63.112	0.004^**^	0.670	0.291	1.506
	CC	51.15	1.615						

^**^Difference is significant at the 0.01 level (2-tailed). ^*^Difference is significant at the 0.05 level (2-tailed).

### 3.3 Participants' emotional resonance with English after intervention

Participants' emotional resonance with English was similarly reassessed after the 8-week period. The findings showed an increase in the EC, with the average score rising from 39.95 to 41.13. The CC, however, demonstrated minimal change (pre-intervention: *M* = 40.18; post-intervention: *M* = 40.31).

The statistical analysis corroborated a significant effect of the pedagogical intervention. An independent samples *t*-test on the post-intervention emotional resonance scores indicated that the EC (*M* = 41.13) scored significantly higher than the CC (41.13 vs. 40.31; *p* = 0.018 < 0.05; Cohen's *d* = 0.549 > 0.5, also reflecting a medium effect size). The analysis confirmed the third hypothesis, indicating that World Englishes education positively enhances emotional resonance with English among Chinese EFL learners.

A detailed comparison of the post-intervention differences in emotional resonance with English between the EC and CC is presented in Table 2.

## 4 Discussion

This quasi-experimental study investigated the English language identity and emotional resonance with English among learners in China through the use of questionnaires. Additionally, it assessed the impact of World Englishes education on these learners' English language identity and emotional resonance. Analysis of the collected data revealed that learners exhibited moderate levels of English language identity and emotional resonance with English before intervention. Furthermore, the findings indicate that World Englishes education positively influences and enhances these levels among learners.

### 4.1 Participants' English identity and emotional resonance of English are at median levels

The analysis indicated that prior to the intervention, participants' average scores for English language identity were 50.89 out of 100 in the EC and 50.87 out of 100 in the CC, both reflecting moderate levels. These results align with findings from previous research (Fong, 2021; Wei, 2016). In the context of China, where English is taught as a foreign language, learners have limited opportunities to use English in daily or professional settings (Yao and Shao, 2024). From the perspectives of both learners and educators, English functions primarily as a communicative tool, with minimal association to cultural dimensions such as emotional connection or a sense of belonging. While proficiency in English may afford learners employment opportunities, economic advantages, or enhanced social status within China, it is not typically regarded as an integral part of their linguistic or cultural identity. Consequently, participants' English language identity remains neither high nor low but rather at a median level.

Regarding emotional resonance with English, the analysis revealed that before the intervention, participants' average scores were 39.95 out of 75 in the EC and 40.18 out of 75 in the CC, indicating relatively low levels. These findings suggest that learners seldom express emotions in English. This contrasts with Caldwell-Harris et al. (2011), who reported that Chinese-English bilinguals residing in the United States, with Chinese as their first language and English as their second, preferred to express emotions in English. Conversely, Chen et al. (2015) found that emotional words in a bilingual's second language tend to have less emotional impact than those in the first language. The present results are consistent with Chen et al. (2015) but contradict Caldwell-Harris et al. (2011). The divergence may be attributed to differences in learning environments and language identity. Participants in Caldwell-Harris et al.'s study lived in an English-speaking environment, frequently used English in daily life, and regarded English as integral to their cultural and social identity, beyond a mere communication tool. In contrast, participants in the current study resided and learned in China, where the Chinese language predominates linguistically, and English remains a foreign language with limited ties to learners' sense of belonging. These contextual factors likely explain why the current findings align with Chen et al. (2015) and diverge from Caldwell-Harris (2014).

### 4.2 World Englishes education improves participants' English language identity

The present study demonstrated that an 8-week instructional intervention in World Englishes significantly enhanced the English language identity of participants in China, as the mean scores of participants' English language identity in the EC was significantly higher than the CC. To the authors' best knowledge, this investigation represents the first experimental effort to effectively influence the English language identity of Chinese university students, thereby contributing a novel academic insight to the field. Identity is conceptualized as “our understanding of who we are and who we think other people are” (Danielewicz, 2001, p. 10). In parallel, language identity pertains to individuals' perceptions of their own language and their views regarding the languages spoken by others. In the Chinese context, English education is categorized as foreign language instruction. Traditionally, Chinese learners have perceived English primarily as the language of Anglo-Saxon cultures. Despite the recognition that proficiency in English facilitates employment opportunities, economic advantages, and social status, learners have generally regarded English merely as a communicative tool, with limited association to their own cultural identity. In contrast, World Englishes education diverges from conventional English teaching by recognizing the equivalence of diverse English varieties, including American English, Australian English, British English, Canadian English, Caribbean English, and Chinese English, and rejecting the notion of English as exclusively the native language of Anglo-Saxon populations. This perspective affirms that any English speaker can legitimately claim ownership of the language. Within this theoretical framework, the current study seeks to elucidate the impact of World Englishes education on fostering learners' English language identity.

### 4.3 World Englishes education improves participants' emotional resonance of English

The findings further indicate that an 8-week World Englishes educational program also fostered greater emotional resonance with the English language among Chinese university students, as the mean scores of participants' emotional resonance with English in the EC was significantly higher than the CC. To the authors' knowledge, this study constitutes the first experimental effort to successfully influence the emotional resonance that Chinese learners have with English, marking another significant academic contribution. As previously discussed, World Englishes education has been identified as a promising avenue for developing learners' English language identity. Supporting this, prior research (Caldwell-Harris et al., 2012) has established that frequent language use contributes to stronger emotional resonance with that language. Theoretically, therefore, World Englishes education holds potential for cultivating learners' emotional engagement with English, a hypothesis confirmed by the current study's results. This educational approach emphasizes fostering learners' awareness of English's linguistic diversity and the intercultural communicative competencies required for effective interaction (Chen, 2022; Rose and Galloway, 2019). By treating different English varieties, including Chinese English, as equal, World Englishes education enhances students' recognition of the diversity among English users and usages, promotes positive attitudes toward non-standard forms, and encourages reflection on the concept of English ownership (Jindapitak et al., 2022; Rajprasit, 2023). Consequently, it strengthens learners' sense of belonging to the English language community. Given that individuals tend to express emotions most authentically in their own language (Weimer et al., 2022), this pedagogical approach effectively deepens learners' emotional resonance with English.

### 4.4 Implication

The present study demonstrates that education in World Englishes can enhance learners' English language identity and their emotional resonance with the language, thereby facilitating more effective English acquisition. Consequently, it is imperative for policymakers and English educators to reconsider their perspectives and actively promote World Englishes education among university students in China.

Firstly, government policymakers must revise existing language and education policies to accord appropriate recognition to English varieties beyond British English and American English. Traditionally, Chinese educational policies have prioritized British English and American English, with the majority of English textbooks reflecting these two varieties. This has led to a widespread perception among English learners in China that British English and American English represent the standard forms, while other English varieties are deemed incorrect. Such attitudes are deeply ingrained within the Chinese linguistic and educational communities. Therefore, it is essential for policymakers to adopt a more inclusive stance by incorporating diverse English varieties, including Chinese English, into educational materials and granting them equal status and legitimacy within the curriculum.

Secondly, university administrators should provide opportunities for English teachers to receive training in World Englishes. Given that the introduction of World Englishes into China is relatively recent, many current English instructors were trained under traditional paradigms and lack sufficient knowledge of World Englishes. Moreover, some educators may hold unfavorable attitudes toward these varieties. It is crucial to cultivate positive perceptions and deepen teachers' understanding of World Englishes. University leadership must thus support professional development initiatives that enable teachers to develop informed, scientific perspectives on World Englishes, acquire relevant knowledge, and gain the pedagogical skills necessary to effectively teach these varieties.

Thirdly, English teachers themselves must revise their attitudes toward World Englishes and acquire both theoretical knowledge and practical teaching methodologies related to this field. As previously noted, many current educators were trained within conventional frameworks and have established fixed views regarding English varieties. They need to adopt more inclusive and updated perspectives on the diversity of English. Simultaneously, it is vital for them to engage in continuous learning about World Englishes and appropriate instructional strategies. Although these adjustments may pose significant challenges, particularly for more experienced teachers, the demonstrated benefits of World Englishes education for students' language acquisition underscore the necessity of these efforts.

### 4.5 Limitation

Upon careful consideration, several limitations of the present study have been identified. First, due to the absence of precise data concerning the number and distribution of university students in China, it was not possible to accurately determine the optimal sample size. As a result, the study employed a sample of 77 university students with a simple random sampling method; however, the extent to which this sample is representative of the broader student population remains uncertain. In future studies, multi-stage sampling methods are recommended to assess more participants to obtain universal research results. Second, the participants were recruited from an educational institution where they demonstrated a high level of motivation for learning, attributable to their involvement in extracurricular educational activities. This elevated enthusiasm may have influenced the training outcomes, thereby limiting the generalizability of the findings to the wider population of students enrolled in formal university programs. Third, although the study proposed several pedagogical recommendations based on its results, these suggestions have not been empirically tested through instructional practice. Consequently, the effectiveness and practical applicability of these recommendations in actual teaching contexts remain undetermined.

## 5 Conclusion

The present quasi-experimental investigation has confirmed that education in World Englishes can enhance Chinese university students' English language identity as well as their emotional resonance with the English language. These findings contribute to the extant literature on World Englishes and English language acquisition within the Chinese context. Nonetheless, the study is subject to certain limitations, notably in terms of sample selection and the lack of exploration of some potential implications. Consequently, the generalizability of the results should be approached with caution. Future research is warranted to address these limitations and to deepen the comprehension of the wider ramifications of World Englishes education.

## Data Availability

The raw data supporting the conclusions of this article will be made available by the authors, without undue reservation.
